# A meta-analysis of inpatient treatment outcomes of severe acute malnutrition and predictors of mortality among under-five children in Ethiopia

**DOI:** 10.1186/s12889-019-7466-x

**Published:** 2019-08-27

**Authors:** Fasil Wagnew, Getenet Dessie, Wubet Worku Takele, Aster Tadesse, Sheikh Mohammed Shariful Islam, Henok Mulugeta, Dessalegn Haile, Ayenew Negesse, Amanuel Alemu Abajobir

**Affiliations:** 1 College of Health Science, Debre Markos University, Debre Markos, Ethiopia; 2 Institute for Physical Activity and Nutrition (IPAN), Deakin University, Melbourne, Australia; 3 College of Health Science, University of Gondar, Gondar, Ethiopia; 4 Faculty of Medicine/school of Public Health, The University of Queensland, Brisbane, Australia; 5 African Population and Health Research Center, Maternal and Child Wellbeing Unit, Nairobi, Kenya; 6 College of Health Sciences, Bahirdar University, Bahirdar, Ethiopia

**Keywords:** Severe acute malnutrition, Treatment outcomes, Meta-analysis, And Ethiopia

## Abstract

**Background:**

Severe forms of malnutrition have drastic effects on childhood morbidity and mortality in sub-Saharan countries, including Ethiopia. Although few studies have previously estimated treatment outcomes of severe acute malnutrition (SAM) in Ethiopia, the findings were widely varied and inconsistent. This study thus aimed to pool estimates of treatment outcomes and identify predictors of mortality among children with SAM in Ethiopia.

**Methods:**

A systematic review was carried out to select 21 eligible articles from identified 1013 studies (dating from 2000 to 2018) that estimated treatment outcomes and predictors of mortality among SAM children. Databases including PubMed, CINHAL, Web of Sciences; Cochrane, Psych INFO and Google Scholar were comprehensively reviewed using medical subject headings (MESH) and a priori set criteria PRISMA guideline was used to systematically review and meta-analyze eligible studies. Details of sample size, magnitude of effect sizes, including Hazard Ratio (HRs) and standard errors were extracted. Random-effects model was used to calculate pooled estimates in Stata/se version-14. Cochran’s Q, I^2^, and meta-bias statistics were assessed for heterogeneity and Egger’s test for publication bias.

**Result:**

Twenty-one studies were included in the final analysis, which comprised 8057 under-five children with SAM in Ethiopia. The pooled estimates of treatment outcomes, in terms of death, recovery, defaulter and transfer out and non-response rates were 10.3% (95% CI: 8.3, 12.3), 70.5% (95% CI: 65.7, 72.2), 13.8% (95% CI: 10.8, 16.9) and 5.1% (95% CI: 3.3, 6.9), respectively. Diarrhea (HR: 1.5, 95% CI: 1.1, 2.2), dehydration (HR: 3.1, 95% CI: 2.3, 4.2) and anemia (HR: 2.2, 95% CI: 1.5, 3.3) were statistically significant predictors of mortality among these children. No publication bias was detected.

**Conclusion:**

Treatment outcomes in under-five children with SAM are lower than the World Health Organization (WHO) standard, where mortality is being predicted by comorbidities at admission. Children with SAM need to be treated for diarrhea, dehydration and anemia at the primary point of care to reduce mortality.

**Electronic supplementary material:**

The online version of this article (10.1186/s12889-019-7466-x) contains supplementary material, which is available to authorized users.

## Background

Globally, an estimated 20 million under-five children suffer from severe acute malnutrition (SAM) contributing to 1 million deaths every year, mainly attributable to comorbid and/or consequent infections [1, 2]. SAM is characterized by two clinical parameters: (1) severe wasting; that is, marasmus, defined as middle upper arm circumference (MUAC) < 115 mm for children aged > 6 months or a weight-for-height < − 3 z-scores according to WHO’s growth standards for under-five children; and (2) nutritional edema with kwashiorkor; that is, the presence of clinically confirmed bilateral, pitting edema [2–4].

Acute malnutrition is a major challenge for achieving sustainable development goals (e.g., Goals 2 and 3–Zero Hunger and Good Health and Wellbeing) as it is associated with major causes of under-five mortality. Moreover, it leads to adverse maternal and child health consequences including retarded school performance and aggravating maternal related problems [5, 6] especially in poorer settings. For instance, 2% of children (nearly 13 million) suffer from SAM in developing countries [7] of which over 90% live in South- East Asia and sub-Saharan Africa [8]. Indeed, SAM is a common indication for pediatric hospital admission and inpatient treatment in these countries. Thus, mortality of children from SAM in inpatient set-ups in sub-Saharan Africa still remains significantly high, with ten-folds higher the risk of death than well-nourished children [9].

Ethiopia has a long history of food insecurity and nutritional disorders aggravated by larger population size, land degradation, and droughts affecting a larger proportion of population [4, 10, 11]. Consequently, the country has been experiencing malnutrition related problems, although both community- and facility-based interventions are in place. For instance, children with SAM had conventionally been managed according to WHO standard in facility set-ups encompassing admission and comprehensive inpatient clinical treatment, although this protocol was first published in 1999 [12] and has guided only inpatient care of complicated SAM patients. Recently (2013 to date), there has been a paradigm shift for treating SAM, using updated WHO treatment guideline published in 2013, with compressive approaches [13]. This includes inpatient, outpatient, and community-based management of acute malnutrition (CMAM) and also provides training for health workers. The main aim of this approach is to decrease over crowdedness by treating non-complicated SAM cases at outpatient treatment programmes (OTP) without admitting to a hospital [7]. Adhering to this protocol has enhanced desired treatment outcomes [14, 15].

In Ethiopia, the health sector has attempted to upgrade nutritional intervention and improve treatment outcomes through health promotion, effective treatment strategy and supplementation of essential micronutrients for children and mothers. Different small scale fragmented studies have been conducted to determine treatment outcomes of children with SAM. However, the evidence base from previous studies on the treatment outcomes of, and factors associated with, SAM are inconsistent, and remain inconclusive. That is, under-five mortality rate in children with SAM ranges from 3.5% [16] to 29% [17] in Ethiopia, depicting a 26% variation between studies. Similarly, recovery rate ranges from 43.5% [16] to 87.6 [18] in different facilities of the country. This suggests that prevention and management of SAM are not uniform and unfinished agendas across the country may be because of lack of access to relevant healthcare, inconsistence use of SAM treatment protocol, etc. In addition, predictors of treatment outcome, particularly mortality, have not been well addressed, although anemia, HIV/AIDS, tuberculosis and diarrhea are reported to be some predictors of time to death [16, 19–23].

The main aim of the current meta-analysis is to determine treatment outcomes and predictors of mortality among under-five children with SAM in Ethiopia. This will assist decision makers and/or other stakeholders to practice effective and efficient SAM management.

## Methods

### Design

This is a systematic review and mata-analysis of published articles on SAM in Ethiopia using a priori *criteria*.

### Search strategies

To identify relevant articles, four authors (FW, GD, HM, and AAA) systematically searched for studies published in English from 2000 to 2018 in PubMed, Web of Science, Cochrane library, Embase, Cumulative Index to Nursing and Allied Health Literature (CINAHL) and Google scholar. Reference lists and grey literature such as programme reports were also retrieved. We used medical subject headings (MESH), adding terms and keywords from a primary search to formulate search strategy in these databases. In all databases, we utilized an interactive process to improve the search strategy through checking numerous search terms and including new search terms as new relevant citations were identified. The keywords included: ‘epidemiology’, ‘treatment outcome’, ‘inpatient severe acute malnutrition’, ‘under-five’, ‘children’, ‘predictors’, ‘associated factors’ and ‘Ethiopia’. Boolean operators – ‘OR’ or ‘AND’ – were used*.* Endnote reference manager software was used to collect and organize search outcomes and for removal of duplicate and /or irrelevant articles.

### Eligibility criteria

Studies conducted in Ethiopia, reporting inpatient treatment outcomes of SAM among under-five children, all published and unpublished observational study designs (i.e., cross-sectional, case-control and cohort) were included. Included studies were within the PICO framework (*P* = under-five children with SAM; I = SAM management; C = under-five children with no selected SAM treatment outcomes; O = under-five children with selected SAM treatment outcomes). Articles with no full text, after email contact with the primary author, and those studies reporting outcomes above the age of 5 were excluded.

### Measurement of outcome variables

The outcomes of interest included the proportion of treatment outcomes of SAM, and predictors of mortality among under-five children with SAM. For predictors, Hazard Ratio (HR) was calculated for dichotomous outcomes from the primary studies. Those predictors included in this study were: sex (‘male’ versus ‘female’), diarrhea (‘no’ versus ‘yes’), dehydration (‘no’ versus ‘yes’), and anemia (‘no’ versus ‘yes’).

### Data extraction

Data extraction format was constructed and pilot-tested with a subset of eligible studies, and then summarized using a table. Two reviewers (FW, HM) independently extracted necessary information from relevant articles. Discrepancies were adjudicated or discussed with a third reviewer (AN), whenever appropriate. We made some efforts to communicate the authors whenever further information was required. Numerator and denominator data and beta coefficients and their standard errors (if given) were used to compute HRs, where HRs with 95% CI were not reported. For dichotomous data, we extracted the number of participants with the outcome and the total sample size. The following study characteristics were extracted: region of the study area, year of publication, participant characteristics, study design, types of hospitals and treatment outcomes.

### Risk of bias

The risk of bias for each relevant article was assessed by two authors (FW and AAA) independently using risk of bias assessment tool. We used the Hoy 2012 addressing internal and external validity tool using 10 criteria [24]. Accordingly, each item has either low or high risk of bias; unclear was categorized as high risk of bias. Overall score of risk of bias was then classified into low, moderate, and high for each eligible study (Additional file 3: Table S1).

### Data processing and analysis

Information about the study design, study sample and country were summarized by Microsoft Excel and then exported to STATA/se version 14 for analysis. Meta-analysis of a pooled proportion of treatment outcomes was carried out using a random-effects model, generating a pooled proportion with 95% CIs. Heterogeneity across studies was estimated using the Cochran’s Q and I^2^ statistics [25]. The I^2^ statistic estimates the percentage of total variations across studies that are due to factual between-study differences rather than chance. We also scrutinized forest plots of summary estimates of each study to determine whether we could identify any heterogeneity between studies. For meta-analyses with a minimum of 10 studies, publication bias was determined based on the visual assessment of the funnel plot [26] and Egger’s test [27].

## Results

### Overview of the search

**Fig. 1:**
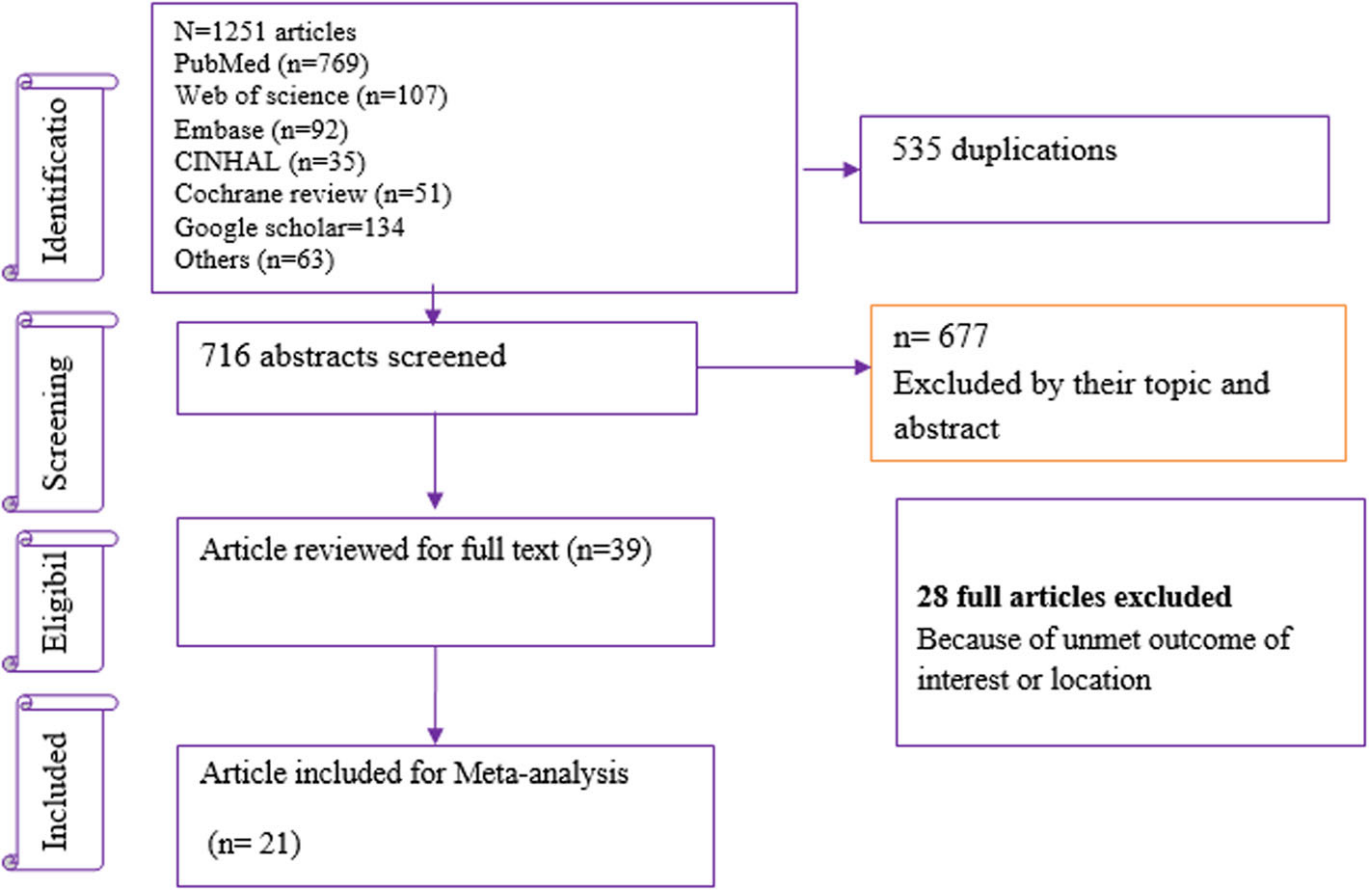
Flowchart diagram describing selection of studies for a meta-analysis of SAM treatment outcomes among inpatient under-five children in Ethiopia

This systematic review and meta-analysis has been reported in accordance with the preferred reporting items for systematic reviews and meta-analyses (PRISMA) statement [28]. First, 1013 articles related to the treatment outcomes of children with SAM were found. Of these, 535 duplications and 667 unrelated articles were excluded. Second, from the remaining 39 potential articles, 21 met eligibility for the review and included in the analysis. Eighteen full-text articles were excluded for the following reasons: 8 articles [29–36] due to unmet outcomes of interest or location and 10 studies [37–46] as they focused on outpatient treatment outcomes (Fig. 1).

Kappa statistic was used to measure agreement between reviewers to determine the uniformity of those potentially eligible full-text articles using the guidelines proposed by Landis and Koch [47]: < 0.20 as slight agreement, 0.21–0.40 fair agreement, 0.41–0.60 moderate agreement, 0.61–0.80 substantial agreement and > 0.80 almost perfect agreement. Finally, the kappa coefficient which revealed the agreement rate between the two reviewers for the included papers was 0.803.

### Descriptive characteristics of the included studies

We identified and included 21 articles with 8057 participants which offered original data on the treatment outcomes of SAM among under-five children in Ethiopia. Main characteristics of the included studies are described in Table 1. These primary studies used different study designs to examine the magnitude of treatment outcomes of SAM. Thirteen out of 21 (61%) studies were retrospective cohort and the remaining 8 (39%) were cross-sectional in design. Sample size ranged from 151 [43] to 947 [48]. Reported under-five mortality rate ranged from 3.5% [16] to 29% [17]. Similarly, the recovery rate ranged from 43.5% [16] to 87.6% [18]. Defaulter rate ranged from 2.6% [49] to 43.6% [16] (Table 1).

**Table 1:** Descriptive summary of 21 included studies on treatment outcomes of SAM among under-five children admitted to a stabilization center in Ethiopia

Authors name	Publication year	Study region	Types of hospital	Study design	Age of participants	Sample size	Death rate (%)	Recovery rate (%)	Defaulter rate (%)	Not recovered (%)
Amsalu S [51]	2006	Amhara	Referral	cross sectional	< 59 months	335	18.2	71.6	8.9	–
G/Michael et al. [20]	2014	Tigray	Referral	cohort	6–59 months	469	12.8	69.7	12.1	5.9
Chane et al. [59]	2014	Amhara	Others	cross sectional	< 59 months	324	6	84.8	4.9	4.0
Ahmed M [82]	2014	Addis Ababa	Referral	cross sectional	6–59 months	193	12.4	75.1	12.4	–
Misganaw C [18]	2014	Oromia	Referral	cross sectional	< 59 months	173	5.7	87.6	6.9	–
Wegen S. et al. [43]	2015	SNNP	Referral	Cross-sectional	< 59 months	151	15.2	69.5	15.2	–
Delelegn Y [58]	2015	SNNP	Others	cohort	6–59 months	420	9.3	82.3	5.7	2.6
Kebede S [17]	2015	Amhara	Others	cohort	< 59 months	415	29	46.9	20.9	2.9
Jarso et al. [48]	2015	Oromia	Referral	cohort	< 59 months	947	9.3	77.8	12.8	–
Tadele, [54]	2016	SNNP	Referral	cohort	< 59 months	449	12.4	–	–	–
Firehiwot [57]	2016	Dire-Dawa	Referral	cohort	< 59 months	617	7.6	69.8	14.2	11.2
Abeje et al. [52]	2016	Amhara	Referral	cross sectional	< 59 months	298	11.7	68.4	19.8	–
Mekuria et al. [22]	2017	Amhara	Others	cohort	6–59 months	253	5.5	77.8	12.2	4.3
Kabeta A [49]	2017	SNNP	Others	cohort	< 59 months	191	16	78.0	2.6	3.1
Wagnew et al. [23]	2017	Amhara	Referral	cohort	< 59 months	527	12.52	67.7	17.8	1.9
Desyalew et al. [55]	2017	Amhara	Referral	cross sectional	6–59 months	401	8.5	76.8	12.9	0.5
Girum, [21]	2017	SNNP	Others	cohort	< 59 months	545	9.3	76.1	4.7	9.7
Admasu A, et al. [56]	2017	SNNP	Referral	cohort	< 59 months	340	8.8	75.5	10	5.6
Tirore et al. [16]	2017	Amhara	Referral	cross sectional	< 59 months	195	3.5	43.5	43.6	9.2
Asres et al. [19]	2018	Amara	Referral	cohort	< 59 months	401	4.2	51.8	35.6	8.2
Behilu T et al. [50]	2018	Amhara	Others	cohort	< 59 months	413	5.8	55.9	16.2	–

SNNP=Southern Nations, Nationalities, and Peoples

### Risk of bias assessment

The risk of bias for each original study was evaluated using toy risk of bias assessment tool which incorporated ten different items [24]. Accordingly, four studies [50–53] had high risk of bias while ten studies [16–23, 48, 49, 54–58] had low risk of bias and the remaining two studies [43, 59] had medium risk of bias (Additional file 3: Table S1). The funnel plot and overall Egger’s test for publication bias revealed no statistically significant evidence, *p*-value = 0.23(Additional file 1: Figure S1).

### Treatment outcomes of under-five children with SAM

**Fig. 2:**
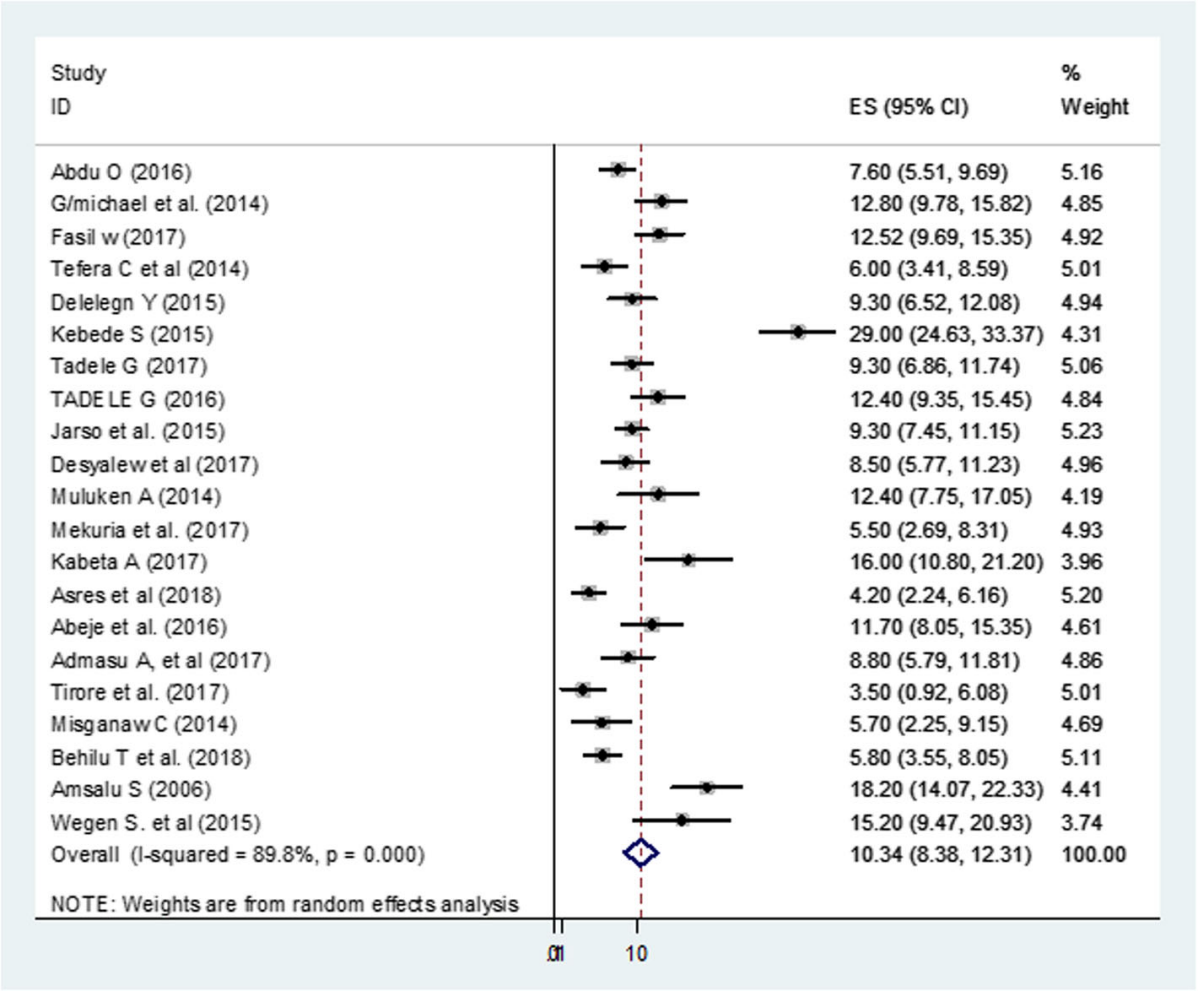
Forest Plot of the 21 studies estimating the mortality rate of under-five children with SAM in Ethiopia

The pooled mortality rate in 8057 under-five children admitted with SAM was 10.3% (95% CI: 8.3, 12.3%) (Fig. 2). On sensitivity analysis, Kebede et al.,2015 [17], and Tirore et al., 2017 [16] had shown an impact on the overall estimation (Additional file 2: Figure S2).

**Fig. 3:**
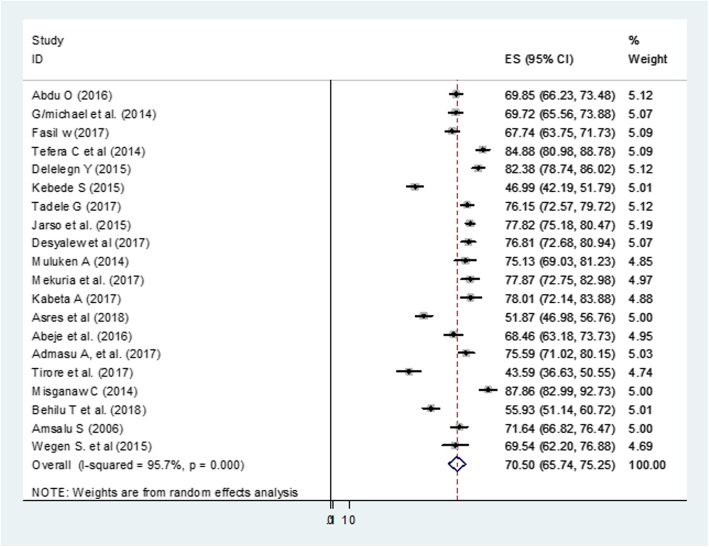
Forest Plot of 20 studies that assessed recovery rate of under-five children with SAM in Ethiopia

The pooled estimate of recovery rate, representing 7608 participants admitted to stabilization centers was 70.5% (95% CI: 65.7, 72.2) (Fig. 3).

**Fig. 4:**
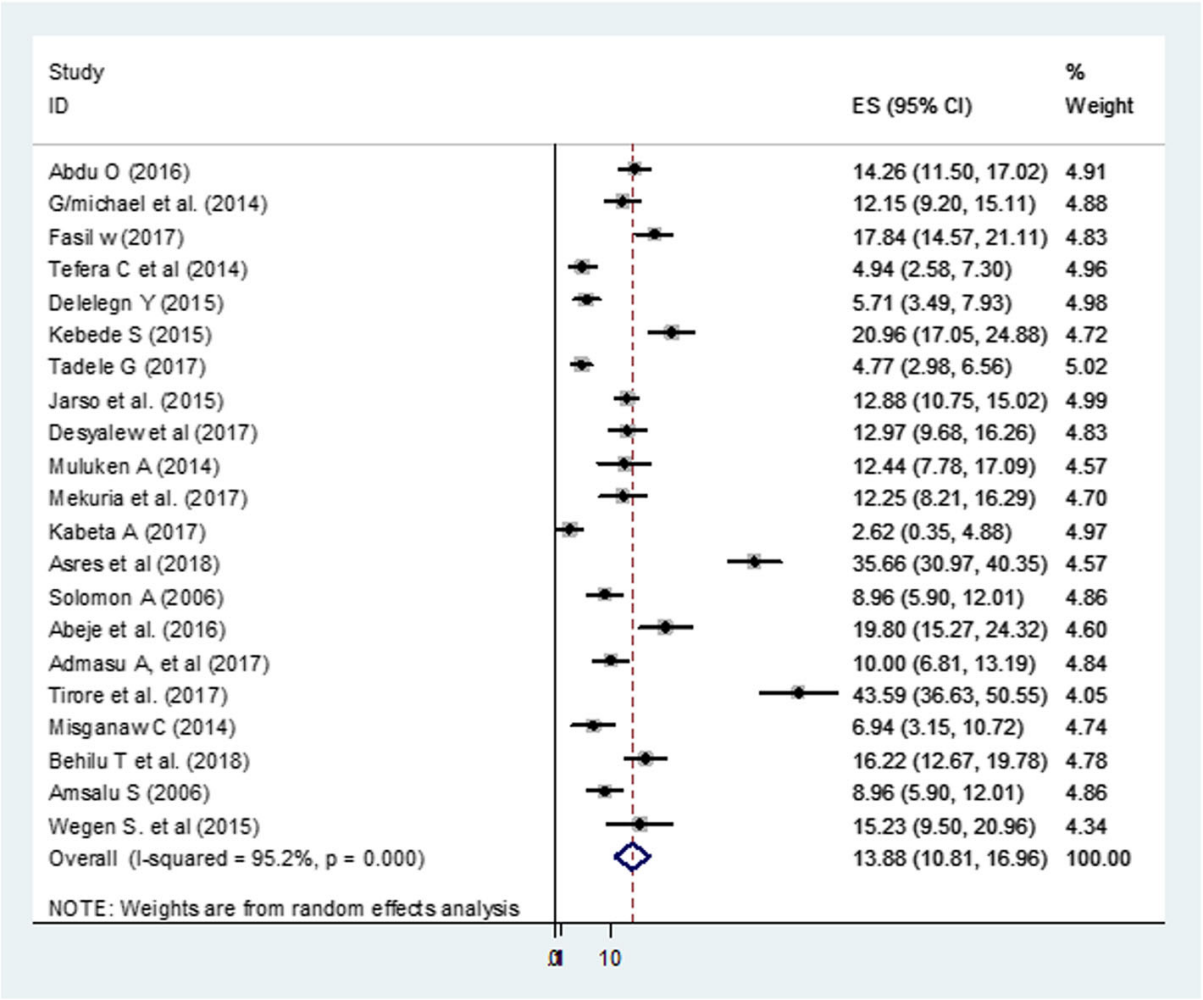
Forest Plot for 21 studies that reported defaulter rate among under-five SAM children in Ethiopia

The pooled estimate of defaulter rate, for 21 studies representing 7943 SAM children admitted to stabilization centers was 13.8% (95% CI: 10.8, 16.9) (Fig. 4).

**Fig. 5:**
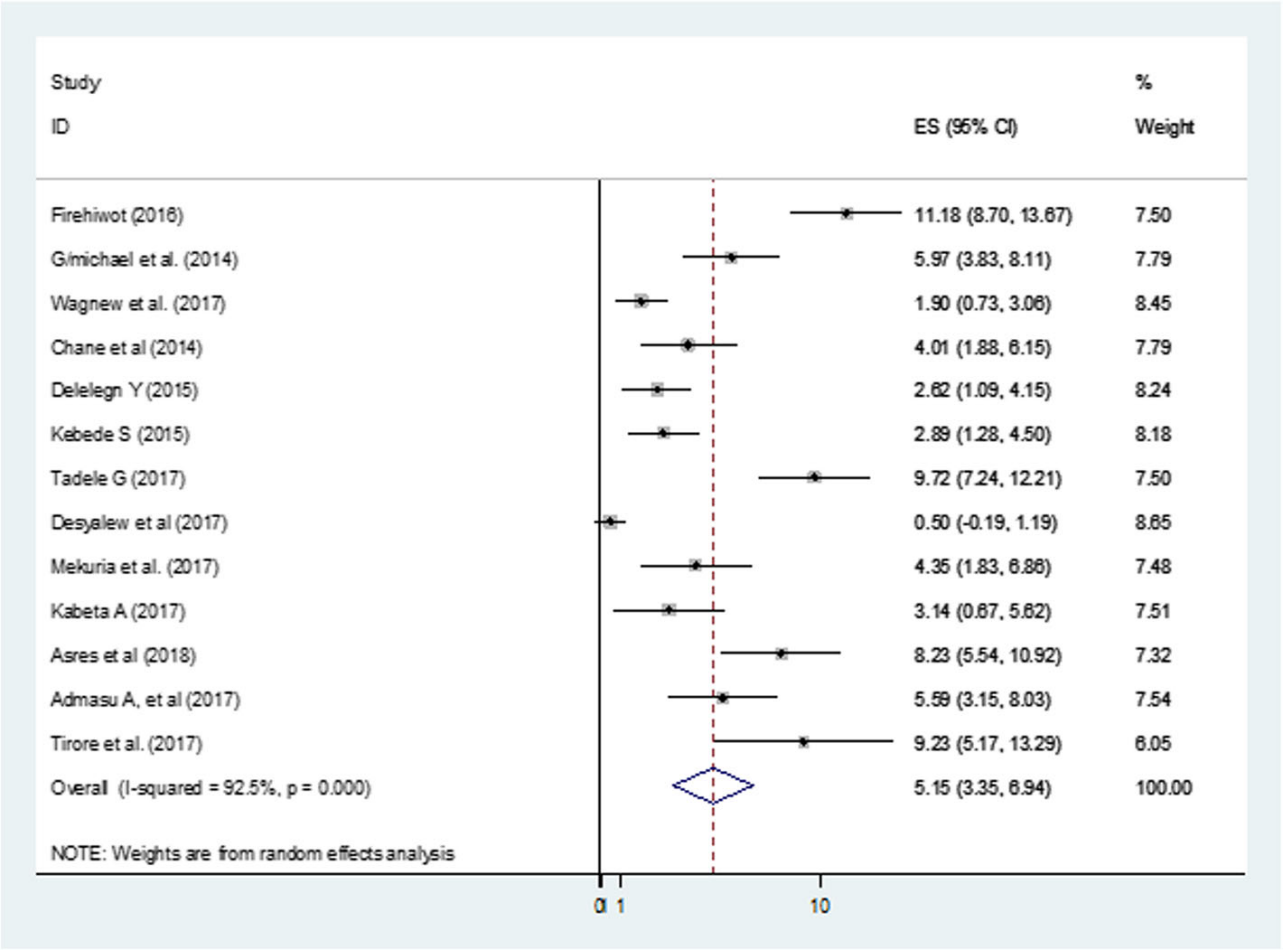
Forest Plot of 13 studies reporting the proportion of non-recovery and transfer out among under-five SAM children in Ethiopia

Finally, the pooled estimate for transfer out and non-response for 13 studies, representing 5098 participants was 5.1% (95% CI: 3.3, 6.9) (Fig. 5).

### Predictors of SAM treatment outcomes

**Fig. 6:**
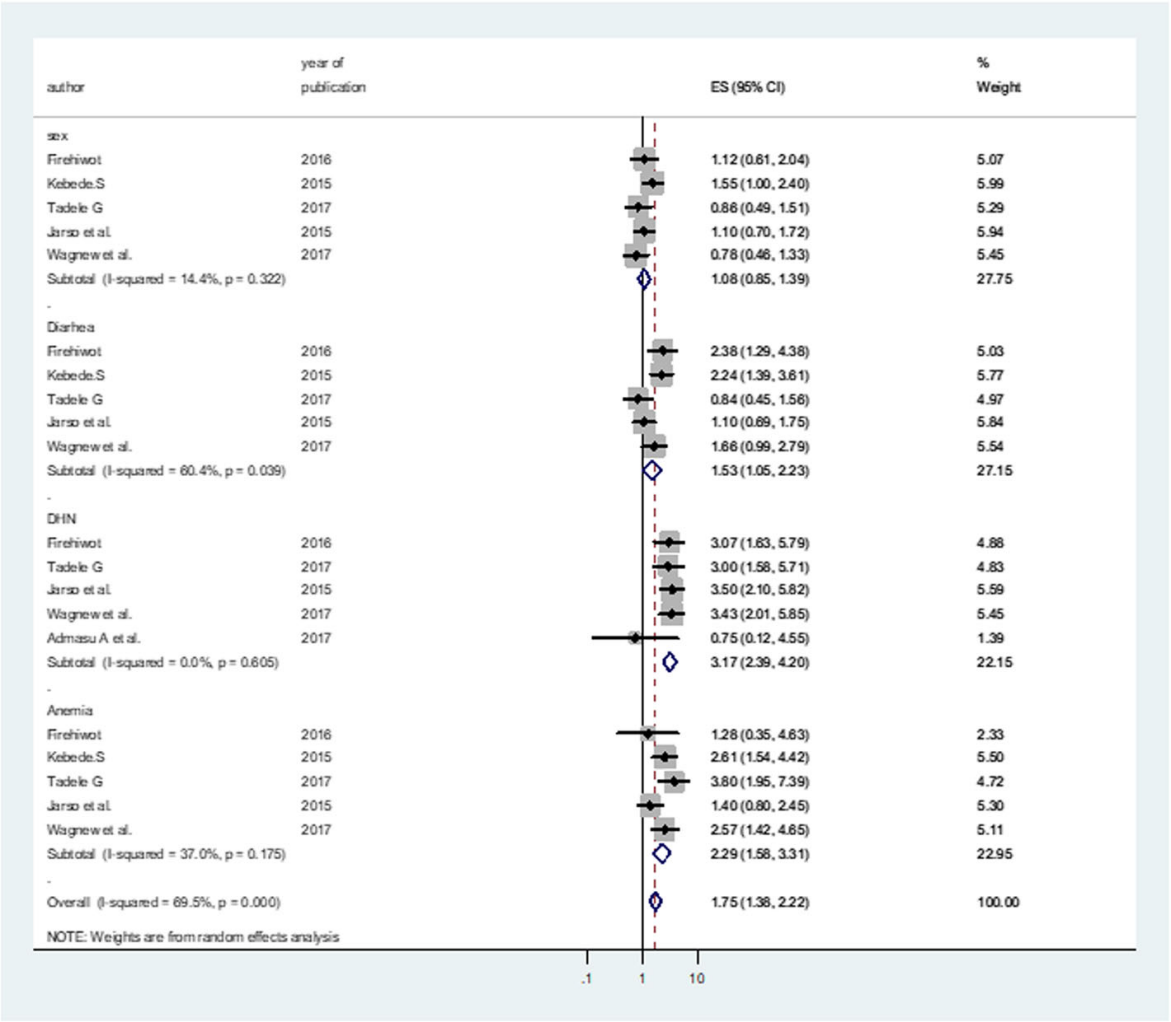
Predictors of mortality among under-five SAM children in Ethiopia

Five studies examined the predictors of time to death among SAM children using adjusted statistical models. Statistically significant factors (*p* < 0.05) associated with time to death were reported. In this analysis, diarrhea, dehydration and anemia were significant predictors of mortality among SAM children. As shown in Fig. 6, the risk of mortality was 1.5 times higher for patients with diarrheal comorbidity as compared to those without diarrheal comorbidity (HR: 1.5 (95% CI: 1.1, 2.2)). Similarly, the likelihood of death was significantly higher in dehydrated patients as compared with those without dehydration (HR: 3.1(95% CI: 2.3, 4.2)).Finally, the risk of death for SAM children with anemia was more than two times higher than those children without anemia (HR: 2.2(95% CI: 1.5, 3.3)) (Fig. 6).

## Discussions

This meta-analysis examined treatment outcomes – death, recovery, defaulter and transfer out plus non-response rates – in under-five children admitted to SAM stabilization centers in Ethiopia. The proportion of children that died and recovered does not achieve the minimum SPHERE standard and WHO management protocol for SAM (i.e., < 10% death rate and > 75%) [60]. This high death rate and low recovery rate could be a result of delay at presentation to a stabilization center, the occurrence of recurrent infections, presence of co-morbidities and non-adherence (by healthcare providers) to the current SAM treatment guideline. The finding was also lower than the previous retrospective review done by Teferi et al. (*n* = 11,335 children) that revealed 87% cure rate and 3.6% (*n* = 468) death rate [15]. Likewise, a study from SierraLeone (*n* = 1987 children) reported 83% recovery rate [61]. This discrepancy might be because of the study characteristics. For instance, previous studies included both moderate and severe forms of acute malnutrition, which might potentially be associated with good treatment outcomes. However, the current analysis reported a recovery rate higher than that reported in Indian study (1130 SAM children) where 51.7% children were discharged from the program upon meeting discharge criteria [62]. Interestingly, the defaulter rate was in parallel with SPHERE report standard stating that the defaulter rate should be less than 15% [60]. By contrast, this finding was lower than a finding in India (47.2% children did not complete their treatment course – defaulters) [62]. The observed variation might be due to sociodemographic characteristics, study period and provision of quality of care, or inefficient use of resources including manpower. Moreover, uncomplicated SAM has occasionally been admitted to Asian stabilization centers [63].

In terms of predictors of mortality in SAM children, the association of diarrhea and SAM is a well-recognized fact [64, 65], although, to the best of our knowledge, the management of children presenting with diarrhea and SAM is limited [66]. This could have a vital role for treatment outcomes. This is supported by several studies [67–71] that have shown increased mortality rates in children who have both diarrhea and SAM comorbidities. This possibly is attributed by the fact that children with diarrhea have concurrent clinical features including severe dehydration, impaired perfusion, and severe metabolic acidosis that would raise mortality. Moreover, gram-negative bacteremia is normally allied with diarrhea and is the major risk factor for death, nonetheless of HIV or anthropometric status [72].

Furthermore, higher mortality rates amongst dehydrated children is likely to be related to the fact that children develop metabolic disturbance and volume deficit with the high prevalence of bacterial disease. Furthermore, the urgent correction of hypotension by speedy volume expansion may attribute to potential sodium and fluid overload that could be a plausible subsidizing factor to death [73–75]. We also found that anemia is one of the predictors responsible for increased mortality in SAM children. Consistently, the risk of earlier death for anemic children is reported to be significantly higher than for children without anemia [14, 76, 77]. The higher risk of mortality is because of an increased prevalence of infection and increased probability of heart failure [78].

Enhancing management of inpatient SAM remains an important approach for reducing malnutrition-related complications including mortality. As such, rendering comprehensive outpatient treatment for most SAM patients may diminish inpatient caseloads, decreases the risks of hospital-acquired infections, and thus, leave staff time to inpatient care. This strategy may reduce case-fatality rates, with cost-effective techniques. Indeed, this is recommended by updated WHO SAM management guideline which is meant to be adhered and implemented in respective set ups.

In general, our findings have policy implications for the management of children with SAM. Policymakers should be cognizant of this to strengthen health systems for appropriate management of SAM. Accordingly, in a bid to improve treatment outcomes for children with SAM, the WHO developed a-ten steps guideline for effective management of SAM, which is a widely accepted standard [79]. Indeed, few studies have reported the feasibility and sustainability of the implementation of this guideline in district hospitals with insufficient resources [80, 81]. Thus, further evaluation studies may fill this gap.

However, the findings need to be considered in the context of some important limitations. These included less control for possible confounders as crude hazard ratios were used to estimate some factors associated with treatment outcomes as opposed to adjusted analyses due to scarcity of data. Moreover, although Egger’s test did not show any risk of publication bias, few studies (e.g., Kebede et al., 2015 and Tirore et al., 2017)) showed high heterogeneity that needs to be considered while interpreting these findings.

## Conclusion

Desired treatment outcomes in under-five children with SAM are lower than the WHO standard, where mortality is being predicted by comorbidities at admission. Healthcare facilities and providers are strongly advised to adhere to and implement based on the up-to-date inpatient SAM treatment and management protocol.

## Additional files

Additional file 1: **Figure S1.** Funnel plots, exploring publication bias for the analysis of pooled estimate (PNG 456 kb)

Additional file 2: **Figure S2.** The sensitivity analysis showed the pooled mortality when the studies omitted step by step (PNG 2054 kb)

Additional file 3: **Table S1.** Risk of Bias assessment Tool of Eligible Articles by using the Hoy 2012 tool (XLSX 13 kb)

## Data Availability

All data generated or analyzed during this study are included within the manuscript [and its supplementary information files].

## Abbreviations

HIV/AIDS: Human Immune deficiency virus/Acquired Immune-deficiency Syndrome
HR: Hazard Ratio
SAM: Severe Acute Malnutrition
WHO: World Health Organization
