# Supplementary figures and images for: A meta-analysis of inpatient treatment outcomes of severe acute malnutrition and predictors of mortality among under-five children in Ethiopia
Source: BMC Public Health. 2019 Aug 27;19:1175. doi: 10.1186/s12889-019-7466-x (PMC6712890; doi:10.1186/s12889-019-7466-x)

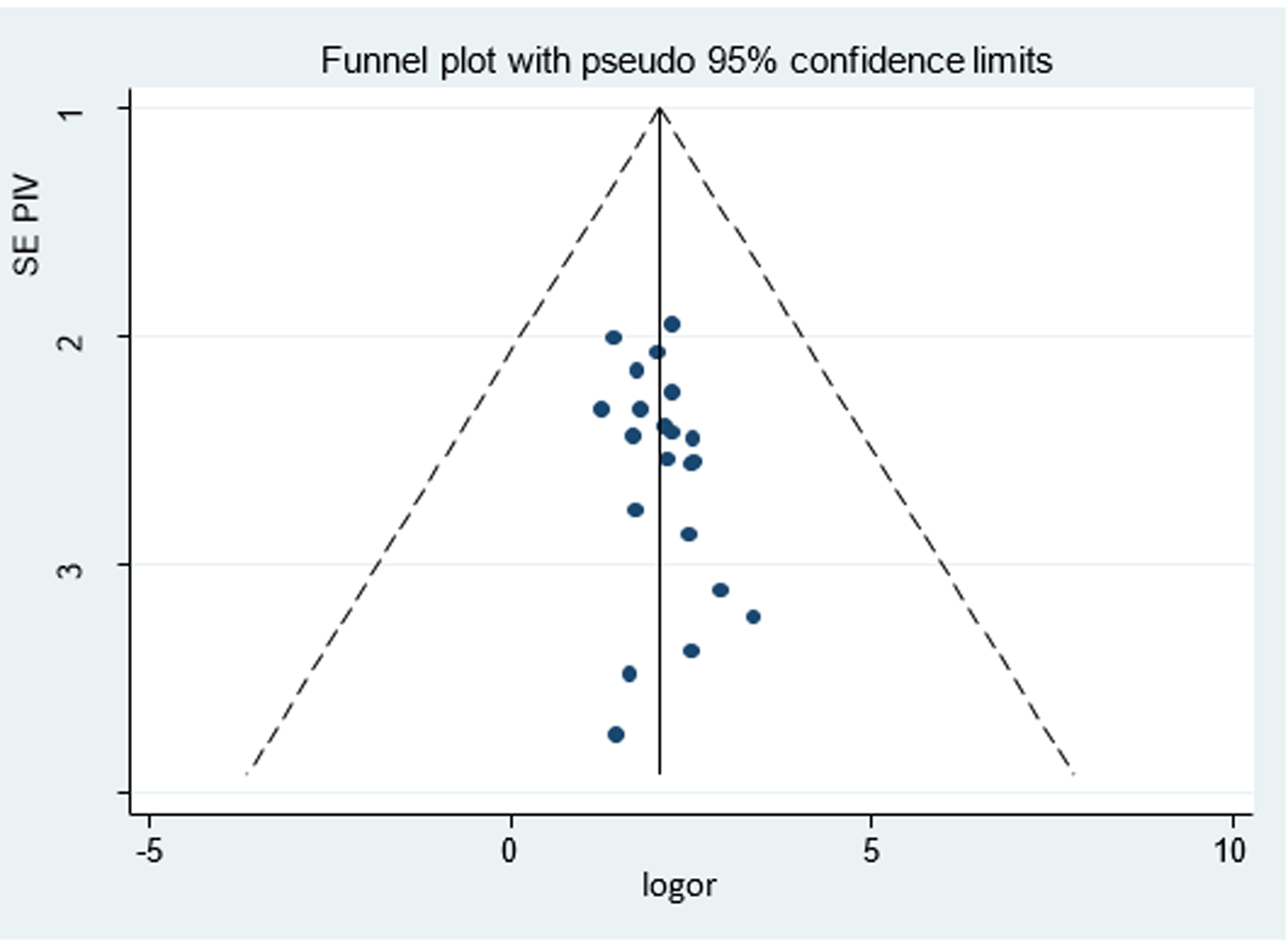

Supplement: Supplementary file 1 — Figure S1. Funnel plots, exploring publication bias for the analysis of pooled estimate (PNG 456 kb) [file 12889_2019_7466_MOESM1_ESM.png]

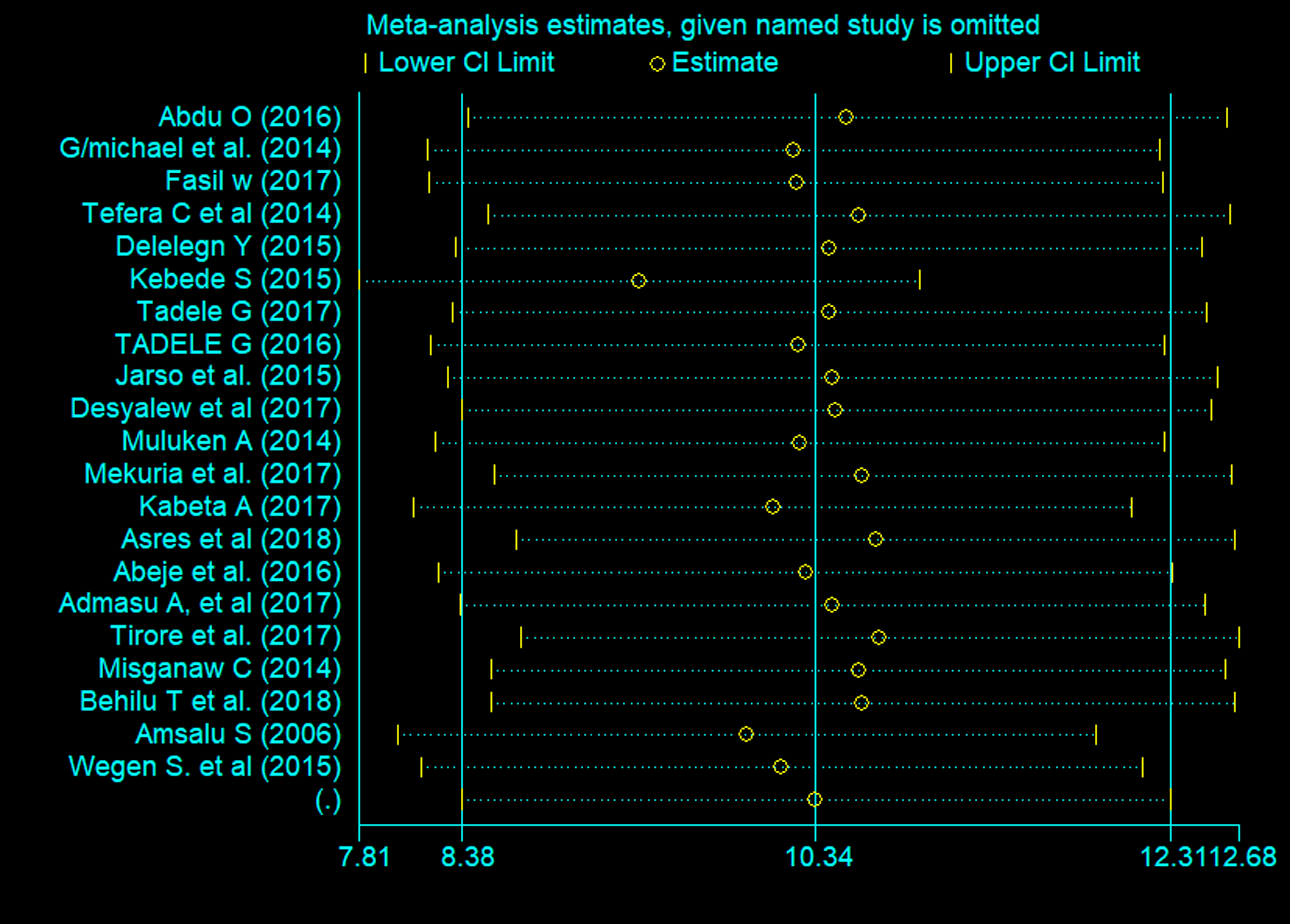

Supplement: Supplementary file 2 — Figure S2. The sensitivity analysis showed the pooled mortality when the studies omitted step by step (PNG 2054 kb) [file 12889_2019_7466_MOESM2_ESM.png]
